# PEYOLO: a wrist fracture detection network based on multi-level receptive field feature extraction and cross-scale fusion

**DOI:** 10.3389/fmed.2026.1850116

**Published:** 2026-05-11

**Authors:** Shuwei Zhang, Rong Tang, Jiong Mu, Shaohai Ren

**Affiliations:** 1 Orthopedic Department, Handan First Hospital, Handan, China; 2 Graduate School, Chengde Medical College, Chengde, China; 3 College of Information Engineering, Sichuan Agricultural University, Ya’an, China

**Keywords:** attention mechanism, deep learning, multi-scale, object detection, wrist fracture

## Abstract

**Introduction:**

The wrist is a high-incidence site for traumatic injuries in the skeletal system, where rapid and accurate diagnosis of fractures is critically important in clinical practice. However, the complex multi-scale variations of fractures impede precise detection of wrist fractures.

**Methods:**

To address this issue, this study proposes a novel wrist fracture detection model, PEYOLO, based on multi-level receptive field feature extraction and cross-scale feature fusion. First, we design a Parallel Dilated Multi-head Attention Module (PDMAM), which performs sparse sampling under different receptive fields to simultaneously obtain receptive fields of varying scales within the same layer. Second, we introduce an Efficient Multi-Scale Attention module, which enhances the perception of multi-scale fracture features by fusing multi-scale spatial information with cross-dimensional dependencies.

**Results:**

Experimental results on the GRAZPEDWRI-DX-fracture dataset demonstrate that PEYOLO improves mAP by 1.4% over the baseline and outperforms several state-of-the-art object detection models.

**Discussion:**

PEYOLO has demonstrated highly advantageous high precision and inference speed, making it an important tool for clinical diagnosis and treatment planning.

## 1 Introduction

The wrist is one of the most frequent sites of traumatic injuries in the human skeletal system, especially among children and the elderly, where the incidence of wrist fractures is significantly high (1). With the widespread use of medical imaging technology, X-ray imaging has become the preferred method for diagnosing wrist fractures (2). However, due to the diversity of fracture types, complex morphology, and issues such as bone over-lap and high background noise in X-ray images, accurate detection of fracture regions re-mains a challenging task (3). Traditional diagnosis relies on manual interpretation by radiologists, which is time-consuming and susceptible to subjective experience, making it difficult to meet the clinical demands for rapid and accurate diagnosis. Moreover, the sharp increase in imaging examinations in recent years has significantly increased the workload of physicians. Emergency physicians often work under high load and high pressure, increasing the risk of missed or misdiagnosed fractures. Therefore, developing efficient and reliable automated computer-aided diagnostic methods is of great value for improving diagnostic accuracy and enhancing the efficiency of clinical workflows in wrist fracture assessment.

With the advancement of artificial intelligence, machine learning and deep learning methods have been widely applied in medical-assisted diagnosis (4). Traditional machine learning methods mainly relied on manually designed features combined with traditional machine learning classifiers. These methods were sensitive to the diversity of target morphology, brightness contrast variations, and had limited generalization capability. In contrast, deep learning models automatically learn highly discriminative feature representations through end-to-end multi-layer neural networks, effectively capturing global and local contextual information of targets, thereby significantly improving model performance and robustness (5).

In recent years, deep learning-based object detection techniques, especially the YOLO series, have shown great potential in medical image analysis (6). The YOLO model uses an end-to-end detection approach to achieve high detection accuracy while maintaining real-time performance, and is widely used in various medical imaging tasks, including the detection of fractures, lung nodules, and cerebral hemorrhages. For wrist fracture detection, X-YOLO introduces a lightweight HGNetV2 backbone and the DySample dynamic upsampling module, reducing the parameter count to 7.0 M while maintaining detection accuracy (7). YOLOv8-AM incorporates a global context (GC) attention mechanism into YOLOv8, effectively improving fracture detection accuracy (8). Although these methods have achieved some progress, they still overlook the multi-scale morphological variations of wrist fractures, which limits detection accuracy.

To address this issue, this study proposes a novel wrist fracture detection model PEYOLO, based on multi-level receptive field feature extraction and cross-scale feature fusion. Using YOLO11n as the baseline, we design the PDMAM that performs sparse sampling under different receptive fields to simultaneously obtain multi-scale features within the same layer. Meanwhile, we introduce the EMA module (9) to enhance the model’s perception of multi-scale fracture features. The contributions of this study are as follows:

(1) We propose the PDMAM module, which achieves multi-level receptive field fea-ture extraction through grouped dilated sampling, improving the model’s adaptability to scale-varying fractures.
(2) We introduce the EMA module, which fuses multi-scale spatial information and cross-dimensional dependencies to enhance the model’s feature representation capability.
(3) We present a novel wrist fracture detection model PEYOLO. Extensive experimental validation on a wrist fracture dataset shows that PEYOLO outperforms advanced models in terms of mAP, demonstrating its effectiveness and robust-ness.

## 2 Related work

### 2.1 YOLO

Object detection is one of the core tasks in computer vision, aiming to locate and identify objects of interest in images (10). In recent years, the YOLO (You Only Look Once) series of models, based on the regression paradigm, has become one of the mainstream frameworks in object detection due to its advantages such as fast detection speed, high accuracy, and ease of deployment (11). Building on the original YOLO (12), YOLOv2 (13) and YOLOv3 (14) introduced anchor boxes, the Darknet-53 backbone, and multi-scale prediction mechanisms, significantly improving small object detection capabilities. Subsequently, YOLOv4 (15) and YOLOv5 (16) systematically integrated CSPNet, Mosaic data augmentation, and other techniques. YOLOv6 (17) introduced self-distillation strategies and efficient hardware-aware network design, providing a paradigm for model light-weighting. YOLOv7 (18) adopted dynamic label assignment and trainable bag-of-freebies strategies, significantly improving model accuracy without increasing inference cost, demonstrating that optimizing the training process alone can bring performance leaps.

Starting with YOLOv8 (19), Ultralytics unified the frameworks for detection, segmentation, pose estimation, and classification, emphasizing modular design and scalability. YOLOv9 (20) addressed the information bottleneck in deep networks by introducing programmable gradient information (PGI) and the generalized efficient layer aggregation network (GELAN). YOLOv10 (21) focused on eliminating inherent post-processing dependencies in detectors, substantially improving end-to-end deployment efficiency through a consistent dual assignment mechanism. Subsequent versions (22–24) further optimized the feature extraction capability of the backbone and the feature pyramid structure, and further improved model accuracy through more refined loss function de-signs and data augmentation strategies.

### 2.2 Fracture detection

Fracture detection is an important research direction in computer-aided diagnosis (CAD) systems (25). Early methods mainly relied on handcrafted features combined with traditional machine learning classifiers to identify fracture regions (26). These methods were sensitive to variations in illumination, angle, and bone morphology, and had limited generalization capability. With the popularity of deep learning, deep learning-based object detection models have gradually become the mainstream approach for fracture detection (27). Some researchers adopted two-stage detectors such as Faster R-CNN (28) for their studies, but the heavy computational cost limits their real-time deployment in emergency scenarios. Other studies used single-stage detectors such as YOLO, which are more advantageous in clinical scenarios with high real-time requirements.

In wrist fracture detection, researchers have conducted extensive explorations based on the YOLO series. Hržić et al. successfully improved fracture detection accuracy in pediatric wrist X-ray images using YOLOv4 (29). X-YOLO introduced a lightweight HGNetV2 backbone and the DySample dynamic upsampling module, reducing the parameter count to 7.0 M while maintaining detection accuracy. YOLOv8-AM incorporated a global context (GC) attention mechanism into YOLOv8, effectively improving fracture detection accuracy. However, existing methods neglect the multi-scale morphological variations of wrist fractures, making it difficult to simultaneously capture both small cracks and large-scale bone morphology. Based on this, we propose the PEYOLO model that combines multi-level receptive fields and cross-scale fusion to further improve the accuracy and robustness of wrist fracture detection.

## 3 Methods

### 3.1 Overview

**Figure 1:**
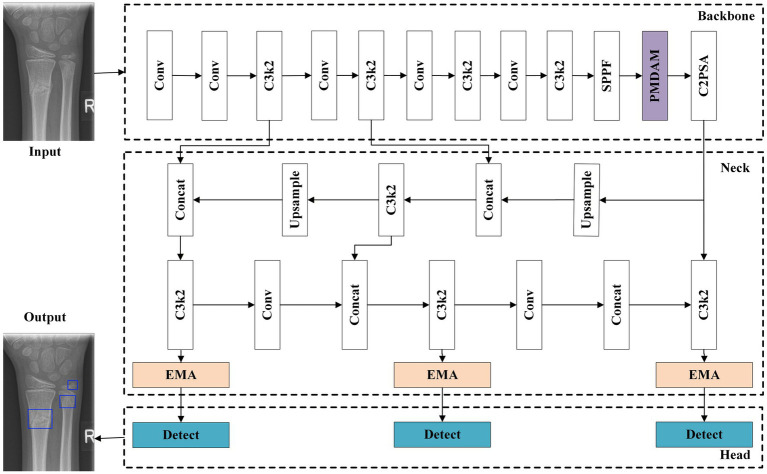
Architecture illustration of our PEYOLO.

PEYOLO uses YOLO11n as the baseline and consists of three parts: backbone, neck, and head. The backbone introduces PDMAM based on YOLOv11n, obtaining multi-level receptive field features by performing sparse sampling under different receptive fields. The neck introduces EMA based on the Feature Pyramid Network (FPN) and Path Aggregation Network (PANet), enhancing the model’s multi-scale perception capability through cross-scale feature fusion. The detection head consists of three detection heads. The net-work architecture is shown in Figure 1.

### 3.2 Parallel dilated multi-head attention module

**Figure 2:**
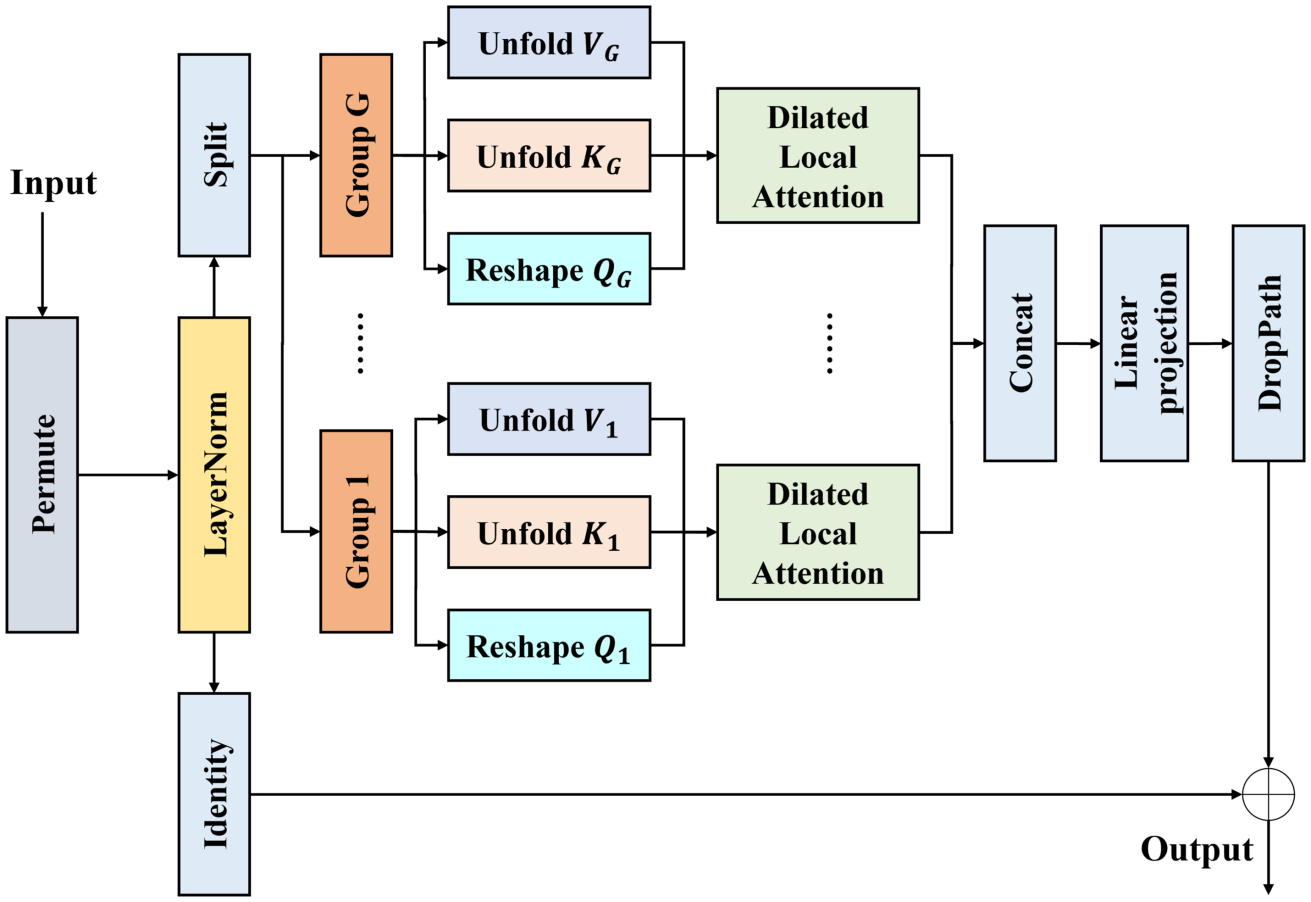
Architecture illustration of our PDMAM.

Fracture detection in wrist X-ray images faces the challenge of large variations in target scales: subtle avulsion fractures may occupy only a few tens of pixels, while comminuted fractures can cover a large area (30). Traditional local attention uses a fixed window size, making it difficult to simultaneously capture small cracks and large-scale bone morphology. Although global attention can cover the entire image, its computational cost grows quadratically with resolution, imposing a heavy computational burden. To address this multi-scale problem, we design the Parallel Dilated Multi-head Attention Module, which performs sparse sampling under different receptive fields to simultaneously obtain receptive fields of varying scales within the same layer. Its network structure is shown in Figure 2.

Given an input feature map F , PDMAM first reshapes the dimensions for convolution operations and then performs layer normalization to obtain Z=LN(F) . Subsequently, Z is fed into the Multi-Head Dilated Local Attention Module (MDLA). MDLA divides the attention heads into G groups, each using a different dilation rate $d_{g}\;(g=1,\ldots ,G)$ , enabling different heads to focus on spatial neighborhoods of different scales. Specifically, the input Z is mapped via 1 × 1 convolution into query, key, and value tensors $[Q,K,V]\in {\mathbb{R}}^{B\times H\times W\times 3C}$ , which are then split into G groups along the channel dimension, yielding $Q_{g},K_{g},V_{g}\in {\mathbb{R}}^{B\times H\times W\times (C/G)}$ . For the $g_{\mathrm{th}}$ group, sparse sampling of keys and values is performed using dilation rate $d_{g}$ and 3 × 3 convolution (Equations 1, 2):


$$K_{g}^{\text{local}}=\text{Unfol}d_{k,d_{g}}(K_{g})$$ (1)

$$V_{g}^{\text{local}}=\text{Unfol}d_{k,d_{g}}(V_{g})$$ (2)

where the Unfold operation flattens the local neighborhood around each position into a vector. Meanwhile, we reshape the query as $Q_{g}\in {\mathbb{R}}^{B\times (C/G)\times 1\times \mathrm{HW}}$ .

Let the dimension of each attention head be $d_{h}=C/N_{h}$ (where $N_{h}$ is the total number of heads). Then the attention output of the $g_{\mathrm{th}}$ group is shown in Equation 3:


$$O_{g}=\text{Softmax}(\frac{Q_{g}^{⊤}K_{g}^{\text{local}}}{\sqrt{d_{h}}})V_{g}^{\text{local}}\in {\mathbb{R}}^{B\times H\times W\times (C/G)}$$ (3)

The outputs of all groups are concatenated along the channel dimension and then linearly projected to obtain the final output of MDLA (Equation 4):


$$\text{MDLA}(Z)=\text{Linear}([O_{1},,,,O_{G}])$$ (4)

Finally, the output of PDMAM is obtained via a stochastic depth residual connection (Equation 5):


$$X_{\text{out}}=X+\text{DropPath}(\text{MDLA}(Z))$$ (5)

Different dilation rates directly control the spatial range sampled for each query position. By computing multiple groups of attention with different dilation rates in parallel, PDMAM simultaneously obtains both fine-grained and coarse-grained feature representations within a single module, effectively alleviating the limitation of fixed receptive fields for detecting scale-varying fracture targets.

### 3.3 Efficient multi-scale attention

**Figure 3:**
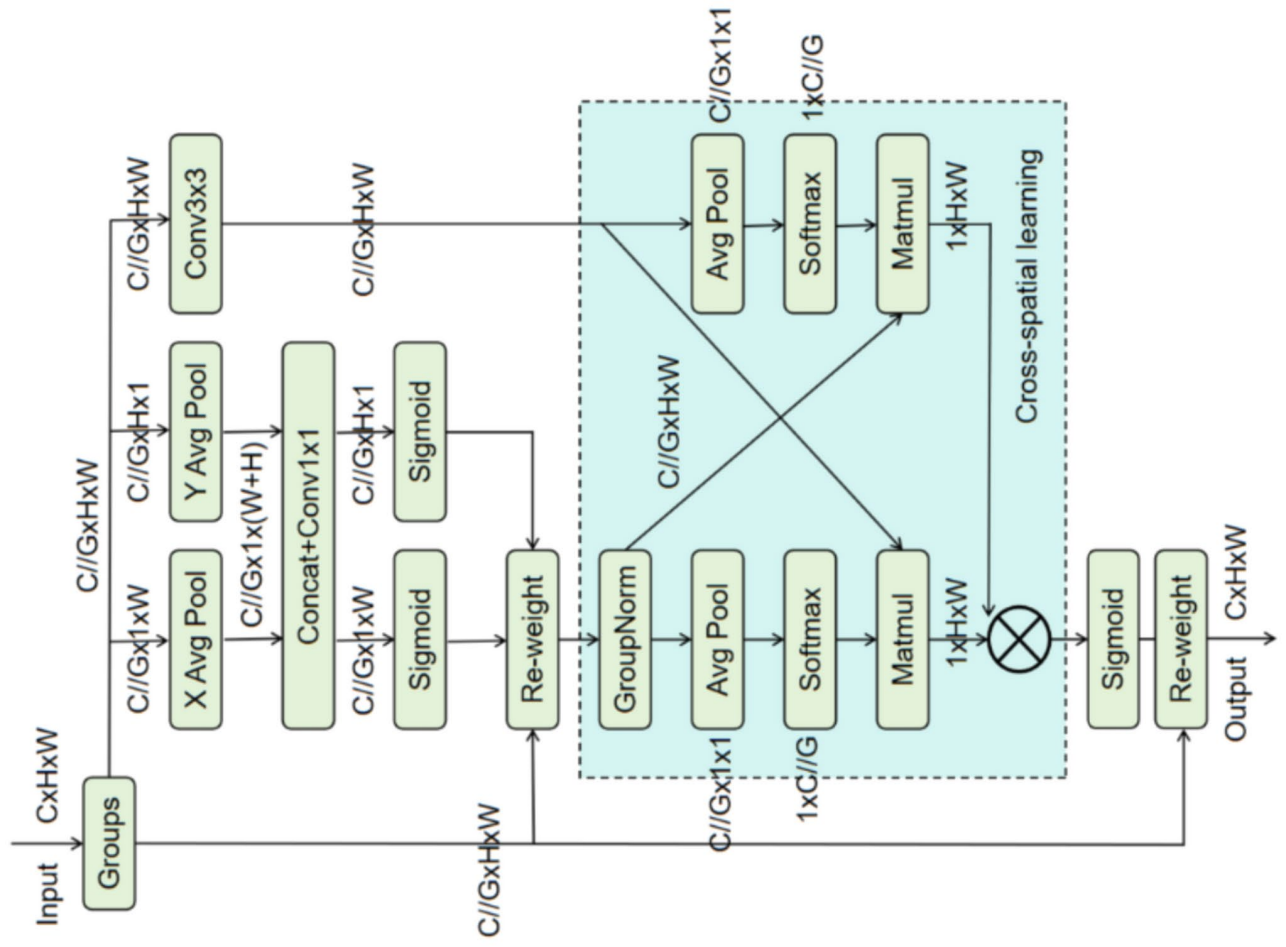
Architecture illustration of EMA.

The morphological presentation of fractures is influenced by multiple factors such as fracture type and degree of displacement, leading to significant differences in the imaging scale of fracture regions (31). This inherent multi-scale characteristic poses a challenge to accurate fracture detection. To this end, this study introduces the EMA module, which enhances the perception of multi-scale fracture features by fusing multi-scale spatial in-formation and cross-dimensional dependencies. Its network structure is shown in Figure 3.

Specifically, for an input feature map $F\in R^{H\times W\times C}$ , EMA first divides it into G sub-features along the channel dimension to learn different semantic information and reduce computational cost. Each sub-feature is then passed through two 1 × 1 convolution branches and one 3 × 3 convolution branch to explore multi-scale spatial dependencies.

In the 1 × 1 branch, one-dimensional global average pooling is performed along the horizontal and vertical directions, respectively, to obtain horizontal and vertical features $z_{c}^{H}$ and $z_{c}^{W}$ . These are concatenated, passed through a 1 × 1 convolution and a Sigmoid activation, generating channel-level attention weights that are used to weight the original features (Equation 6):


$$F_{R}=\delta (\mathrm{Con}v_{1\times 1}(\text{Concat}(z_{c}^{H},z_{c}^{W})))⊗F$$ (6)

where σ denotes the Sigmoid activation function, Concat denotes feature concatenation, and ⊗ denotes element-wise multiplication.

In the 3 × 3 branch, EMA captures local cross-channel interactions, and the output feature is shown in Equation 7:


$$F_{\text{Conv}3\times 3}=\mathrm{Con}v_{3\times 3}(F)$$ (7)

Subsequently, a cross-spatial learning stage follows. Global average pooling is applied to the 1 × 1 branch enhanced features $F_{R}$ and the 3 × 3 branch features $F_{\text{Conv}3\times 3}$ to fuse long-range and local multi-scale context information. The calculation process is shown in Equations 8 and 9:


$$z_{1}=\frac{1}{H\times W}\sum_{j}^{H}\;\sum_{i}^{W}F_{R}(i,j)$$ (8)

$$z_{3}=\frac{1}{H\times W}\sum_{j}^{H}\;\sum_{i}^{W}F_{\text{Conv}3\times 3}(i,j)$$ (9)

where $z_{1}$ and $z_{3}$ are the global average pooling outputs of the 1 × 1 and 3 × 3 branches, respectively.

After applying Softmax to the pooled results, matrix multiplication is performed to generate two pixelwise attention maps, which are then element-wise multiplied, followed by a Sigmoid activation to obtain spatial attention weights. Finally, the attention weights are element-wise multiplied with the original grouped features to complete spatial enhancement, yielding the output (Equation 10):


$$\text{Out}=\delta (\delta (z_{1})\times \delta (z_{3}))⊗F$$ (10)

By capturing spatial features at different scales and fusing multi-scale spatial infor-mation, EMA effectively enhances the model’s perception capability for multi-scale targets.

## 4 Results and discussion

### 4.1 Experiments setup

To ensure the fairness of all comparative experiments and the reproducibility of the results, this section details the hardware platform, software environment, and specific training parameters used for all models.

#### 4.1.1 Hardware and frameworks

The experimental platform employs an NVIDIA GeForce RTX 4090 (24 GB) GPU. All experiments are implemented using Python 3.8.10 and the PyTorch 2.0.0 (cu118) deep learning library. To ensure that all compared models are evaluated under identical training procedures, the Ultralytics 8.3.160 code framework is uniformly adopted for all experiments.

#### 4.1.2 Training parameters

All models involved in the experiments are trained with consistent hyperparameters. In terms of training strategy, each model is trained from scratch without loading any pre-trained model from external datasets. The batch size is set to 16, and the number of training epochs is 100. All models are trained using the AdamW optimizer, with an initial learning rate of 0.002 and a momentum of 0.9. In our implementation of PDMAM, the total number of attention heads is set to 4. These heads are divided into G = 2 groups based on dilation rate, with each group containing 2 heads. The dilation rates for the two groups are set to d∈{1,2} , enabling the model to capture both local fine-grained features (dilation = 1) and larger contextual patterns (dilation = 2). The unfolding kernel size is k = 3. The DropPath stochastic depth rate is set to 0.1. In EMA, G = 8.

### 4.2 Dataset

The GRAZPEDWRI-DX (32) dataset is a collection of X-ray images of pediatric wrist injuries, comprising 20,327 images from 6,091 patients collected at the University Hospital Graz in Austria between 2008 and 2018. As this study focuses on wrist fractures, we selected all fracture images from the GRAZPEDWRI-DX dataset to create the GRAZPEDWRI-DX-fracture dataset.

The GRAZPEDWRI-DX-fracture dataset comprises 13,550 images. To prevent data leakage, we performed a patient-level split. Specifically, images were sorted by patient ID (the first four digits of the filename). We then assigned 70% of patients (9,485 images) to the training set, 10% (1,355 images) to the validation set, and 20% (2,710 images) to the test set.

### 4.3 Evaluation metric

Following the convention in the object detection field, this study adopts Precision (P), Recall (R), and mean Average Precision (mAP) as the evaluation metrics to comprehensively and objectively assess the performance of PEYOLO and all compared models.

Precision measures, among the samples predicted as positive by the model, how many are true positives. Recall measures, among all actual positive samples, how many are successfully predicted by the model. Their calculation formulas are shown in Equations 11 and 12:


$$\text{Precision}=\frac{\mathrm{TP}}{\mathrm{TP}+\mathrm{FP}}$$ (11)

$$\text{Recall}=\frac{\mathrm{TP}}{\mathrm{TP}+\mathrm{FN}}$$ (12)

where TP (True Positive) denotes correctly detected targets, FP (False Positive) denotes false detections (background recognized as a target), and FN (False Negative) denotes missed detections (real targets not detected).

mAP50 is the core metric for evaluating the overall performance of a model, defined as the mAP value when the IoU (Intersection over Union) threshold is fixed at 0.5. It directly reflects the detection capability of the model.

### 4.4 Comparison experiments

To comprehensively evaluate the performance of the proposed PEYOLO model, we compared it with several state-of-the-art YOLO-based object detectors, including YOLOv5, YOLOv6, YOLOv8, YOLOv9, YOLOv10, and the baseline YOLO11. All models were trained and tested under identical experimental settings on the GRAZPEDWRI-DX-fracture dataset. Tables 1, 2 report the quantitative results on the validation set and test set, respectively.

**Table 1:** Comparison with the advanced methods on the validation set.

Model	Precision	Recall	mAP
YOLOv5	91.3	85.9	93.4
YOLOv6	91.1	88.3	94.2
YOLOv8	91.7	86.6	93.8
YOLOv9	92.5	87.1	94.0
YOLOv10	92.8	85.1	93.4
YOLO11	91.3	86.1	93.6
YOLOv8ResCBAM (33)	91.6	87.5	94.4
YOLOv8-AM	91.9	87.2	94.5
Gold-YOLO (34)	91.8	88.2	93.9
PEYOLO	91.5	89.2	94.8

**Table 2:** Comparison with the advanced methods on the test set.

Model	Precision	Recall	mAP
YOLOv5	92.0	86.3	93.8
YOLOv6	91.9	88.6	94.1
YOLOv8	92.6	87.8	94.4
YOLOv9	92.2	88.5	94.5
YOLOv10	92.5	86.8	94.0
YOLO11	91.8	88.2	94.1
YOLOv8ResCBAM	93.1	88.3	95.1
YOLOv8-AM	93.0	88.4	95.0
Gold-YOLO	92.1	88.4	94.4
PEYOLO	93.3	89.0	95.5

As shown in Table 1, PEYOLO achieves the highest recall (89.2%) and mAP (94.8%) among all compared methods on the validation set. Compared to the baseline YOLO11, PEYOLO improves recall by 3.1 percentage points and mAP by 1.2 percentage points, demonstrating its superior ability to detect true positive fracture instances. This improvement of Recall corresponds to approximately 31 fewer missed fractures per 1,000 wrist X-rays compared to the baseline YOLO11 model. Although YOLOv10 achieves the highest precision (92.8%), its recall is relatively low (85.1%), indicating a more conservative detection strategy that may miss some fractures. Notably, YOLOv8ResCBAM and YOLOv8-AM also show competitive performance, achieving mAP values of 94.4 and 94.5%, respectively, yet they still fall short of PEYOLO in recall and overall mAP. Gold-YOLO, while achieving a recall of 88.2%, lags behind in mAP (93.9%). In contrast, PEYOLO strikes a better balance between precision and recall.

On the test set (Table 2), PEYOLO again outperforms all other models, achieving the highest precision (93.3%), recall (89.0%), and mAP (95.5%). Compared to YOLO11, PEYOLO improves precision by 1.5%, recall by 0.8%, and mAP by 1.4%. Among the state-of-the-art methods, YOLOv8ResCBAM and YOLOv8-AM also yield competitive results, with mAP values of 95.1 and 95.0%, respectively, and both achieve over 93% precision. However, their performance metrics were all lower than those of PEYOLO. Although Gold-YOLO maintains a reasonable recall rate of 88.4%, it lags behind in terms of mAP and precision. These results consistently indicate that the proposed PDMAM and EMA modules effectively enhance the model’s ability to handle multi-scale fractures, leading to better detection accuracy and robustness.

To more comprehensively demonstrate the practical value of the proposed method in actual clinical settings, we evaluated the model parameters and inference time before and after the improvement, with the results shown in Table 3. The results indicate that the improved PEYOLO model has only 2.8 million parameters and an inference time of 7.1 ms, representing an increase of just 0.1 million parameters and 0.3 ms compared to the baseline. Although there is a slight increase in parameters and inference speed, given the significant improvement in detection accuracy achieved by PEYOLO, this minimal computational cost remains within clinically acceptable limits. These results demonstrate that the proposed improvements enhance the model’s performance in clinical settings without unduly increasing model complexity, indicating strong practicality and deployment potential.

**Table 3:** Comparison of model size and inference speed.

Model	Parameter	Inference time
YOLO11	2.7 M	6.8 ms
PEYOLO	2.8 M	7.1 ms

To assess the stability and statistical significance of our results, we repeated the experiments three times for both PEYOLO and the suboptimal model (YOLOv8-AM). Table 4 reports the mean and standard deviation for each metric. On the validation set, PEYOLO achieves a mean mAP of 94.87% with a standard deviation of 0.06, consistently outperforming YOLOv8-AM. On the test set, PEYOLO achieves a mean mAP of 95.47% with a standard deviation of 0.06%, consistently outperforming YOLOv8-AM (94.97% ± 0.25%). An independent two-sample t-test comparing the test set mAP values between PEYOLO and YOLOv8-AM yielded a *p*-value<0.05, indicating that the improvement is statistically significant. The small standard deviations across all metrics demonstrate that our proposed modules provide stable and reliable performance gains.

**Table 4:** Multiple experimental comparisons with the suboptimal model.

Model	Precision_v	Recall_v	mAP_v	Precision_t	Recall_t	mAP_t	Parameters
YOLOv8-AM (1)	91.9	87.2	94.5	93.0	88.4	95.0	3.0 M
YOLOv8-AM (2)	92.3	88.8	94.6	93.0	89.5	95.2	3.0 M
YOLOv8-AM (3)	91.8	88.3	94.4	92.7	88.6	94.7	3.0 M
YOLOv8-AM	92.00 ± 0.26	88.10 ± 0.82	94.50 ± 0.1	92.90 ± 0.17	88.83 ± 0.59	94.97 ± 0.25	3.0 M
PEYOLO (1)	91.5	89.2	94.8	93.3	89.0	95.5	2.8 M
PEYOLO (2)	91.9	88.9	94.9	92.5	90.0	95.5	2.8 M
PEYOLO (3)	91.9	88.3	94.9	92.7	89.3	95.4	2.8 M
PEYOLO	91.77 ± 0.23	88.80 ± 0.46	94.87 ± 0.06	92.83 ± 0.42	89.43 ± 0.51	95.47 ± 0.06	2.8 M

**Figure 4:**
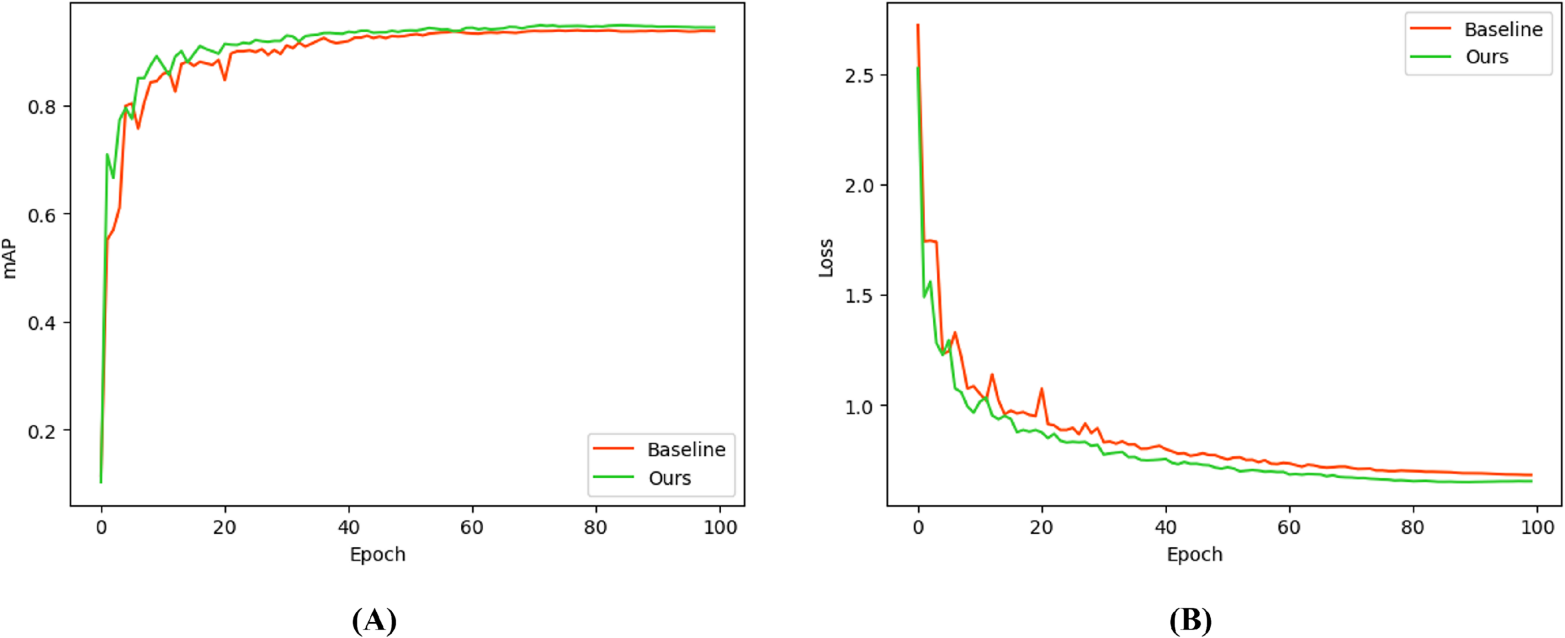
Comparison of training processes. **(A)** shows the mAP comparison, and **(B)** shows the loss comparison.

Figure 4 presents the mAP curves and loss curves of PEYOLO and the baseline YO-LO11 during training. As shown in Figure 4A, the mAP of PEYOLO remains consistently higher than that of the baseline throughout the entire training process, with faster convergence. Notably, during the first 20 epochs, the mAP of PEYOLO rises significantly faster than that of the baseline, indicating that the PDMAM and EMA modules effectively accelerate the model’s adaptation to multi-scale fracture features. From the loss curves in Figure 4B, both the training loss and validation loss of PEYOLO decrease to lower values, demonstrating that the proposed modules possess strong generalization capability.

### 4.5 Ablation study

To further demonstrate the contributions of each module, we conducted ablation ex-periments on the validation and test sets. The experimental results are presented in Tables 3, 4.

Table 5 shows the ablation results on the validation set. Introducing the EMA module alone significantly increased the recall on the validation set from 86.1 to 88.3%, and the mAP from 93.6 to 94.4%. This indicates that EMA effectively enhances the model’s sen-sitivity to fracture regions through cross-spatial learning and multi-scale information fu-sion. Specifically, the horizontal and vertical global pooling in the 1 × 1 branch of the EMA module captures long-range dependencies, while the 3 × 3 branch preserves local details; the synergistic effect of the two branches significantly improves the discriminative ability of the features. After further adding the PDMAM module on top of EMA, the recall continued to increase from 88.3 to 89.2%, and the mAP from 94.4 to 94.8%. By employing multiple attention heads with different dilation rates, PDMAM enables the model to simultaneously focus on both small-scale cracks and large-scale bone morphology, thereby further reducing missed detections of multi-scale fractures. Notably, the precision remained unchanged after adding PDMAM, indicating that this module expands the receptive field without increasing the risk of false detections, demonstrating the robustness of its design.

**Table 5:** Results of ablation experiments on the validation set.

Model	EMA	PDMAM	Precision	Recall	mAP
Baseline	×	×	91.3	86.1	93.6
+EMA	√	×	91.5	88.3	94.4
PEYOLO	√	√	91.5	89.2	94.8

Table 6 presents the ablation results on the test set. Introducing the EMA module alone significantly increased the precision on the test set from 91.8 to 92.7%, the recall from 88.2 to 88.6%, and the mAP from 94.1 to 94.9%. The overall improvement across all metrics further confirms the effectiveness of EMA. On this basis, the addition of the PDMAM module brought continued marginal gains. The precision, recall, and mAP of the model further improved to 93.3, 89.0, and 95.5%, respectively. The test set results again confirm that through the multi-receptive-field sensing mechanism, PDMAM enables the model to simultaneously attend to bone morphologies at different scales, thereby significantly improving the detection accuracy for multi-scale fracture targets.

**Table 6:** Results of ablation experiments on the test set.

Model	EMA	PDMAM	Precision	Recall	mAP
Baseline	×	×	91.8	88.2	94.1
+EMA	√	×	92.7	88.6	94.9
PEYOLO	√	√	93.3	89.0	95.5

### 4.6 Visual comparison

**Figure 5:**
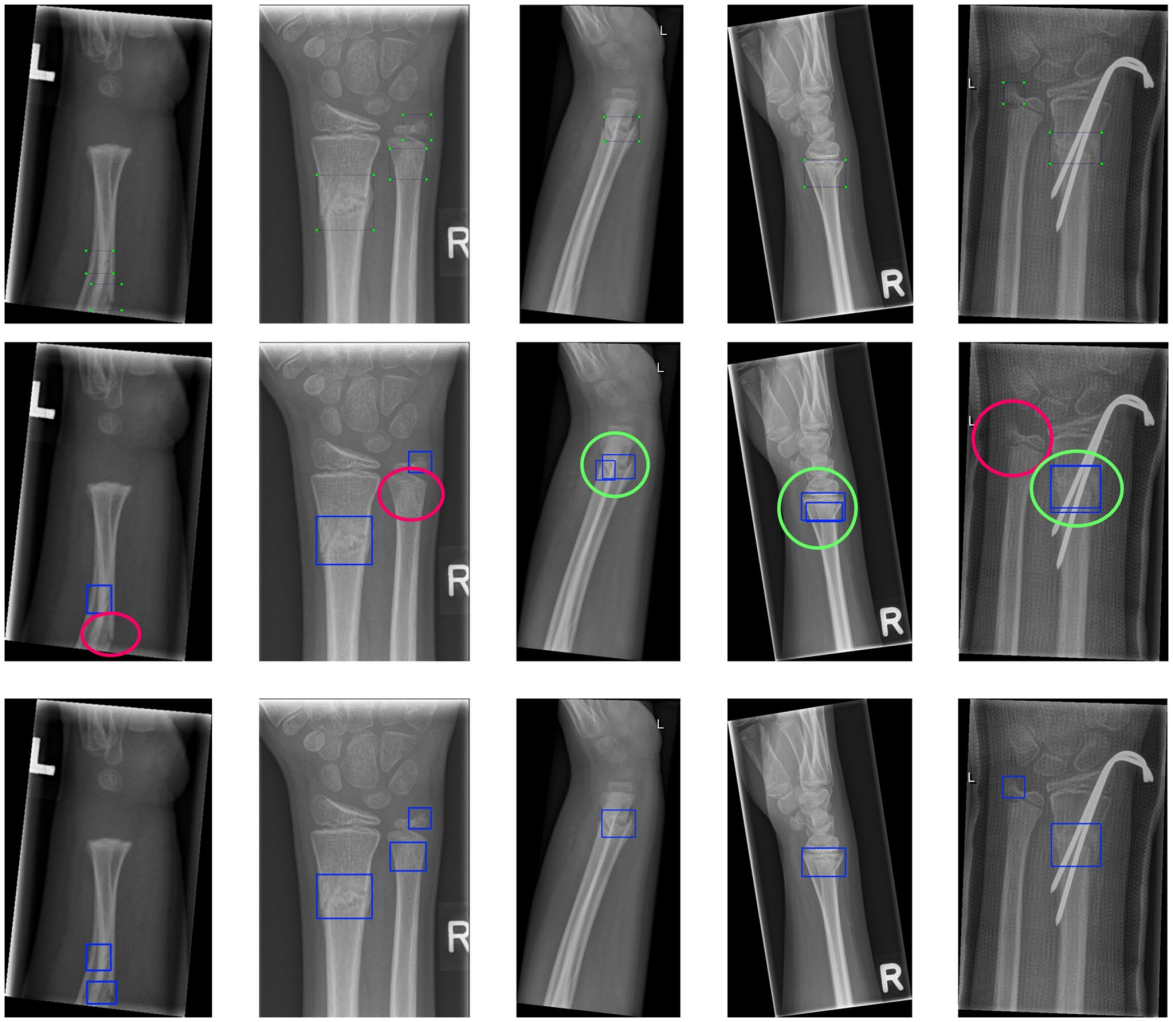
Visual comparison of detection results. The top row is the label. The middle row is Baseline, the bottom row is PEYOLO. The neon green circle represents wrong detection, and the magenta circle represents missed detection.

Figure 5 presents a visual comparison of detection results between PEYOLO and the baseline model on representative wrist X-ray images, where yellow circles indicate false positives and red circles indicate missed detections. Compared with the baseline, PEYOLO can effectively distinguish multi-scale fracture targets and significantly reduces missed detections and false positives on difficult samples. The qualitative results fully demonstrate that the introduction of EMA and PDMAM effectively enhances the model’s multi-scale feature perception capability, exhibiting excellent robustness and clinical utility in challenging scenarios.

### 4.7 Limitations and future directions

Although PEYOLO achieves promising results across multiple metrics, it still has certain limitations.

First, there remains room for improvement in model precision, recall, and mAP. Future work could further optimize these metrics by incorporating super-resolution or feature enhancement modules.

Second, this study only utilizes a single dataset. Although this dataset is relatively large, the limitations in image acquisition equipment, exposure parameters, and patient population may affect the model’s generalization ability in other clinical settings. Future work should conduct cross-domain validation on multi-center, multi-device, and different age-group datasets to evaluate the robustness of the model.

Third, the current work relies solely on X-ray images. In the future, exploring multi-modal fusion by integrating clinical textual information (e.g., patient age, trauma mechanism) or other imaging modalities (e.g., CT, MRI) with X-ray images could further improve the accuracy of fracture detection.

## 5 Conclusion

This paper proposes a novel object detection network, PEYOLO, for wrist fracture de-tection. Using YOLO11n as the baseline, the method designs a Parallel Dilated Multi-head Attention Module (PDMAM) to effectively address the large scale variation of fracture tar-gets through sparse sampling under multiple receptive fields. In addition, the Efficient Multi-Scale Attention (EMA) module is introduced to enhance the model’s ability to fuse multi-scale spatial information. Experimental results on the GRAZPEDWRI-DX-fracture dataset demonstrate that PEYOLO outperforms several advanced models in terms of Precision, Recall, and mAP, demonstrating strong detection performance and clinical utility. Future work will focus on model lightweighting, cross-disease transfer learning, and multimodal image fusion.

## Data Availability

The original contributions presented in the study are included in the article/supplementary material, further inquiries can be directed to the corresponding author.
